# Progress Toward Measles Elimination — World Health Organization Eastern Mediterranean Region, 2019–2022

**DOI:** 10.15585/mmwr.mm7307a1

**Published:** 2024-02-22

**Authors:** Nasrin Musa, Amany Ghoniem, Christopher H. Hsu, Sondos Mubarak, Gerald Sume, Mohammed Sharifuzzaman, JeongEun Bak, Yvan Hutin, Nadia Teleb, Natasha Crowcroft, Patrick O’Connor, Frank Mahoney, Quamrul Hassan

**Affiliations:** ^1^Immunization Vaccine Preventable Disease and Polio Transition Unit, Department of Communicable Diseases, Regional Office of the Eastern Mediterranean, World Health Organization, Cairo, Egypt; ^2^Department of Immunization, Vaccines, and Biologicals, World Health Organization, Geneva, Switzerland; ^3^Global Immunization Division, Center for Global Health, CDC.

**Summary**

**What is already known about this topic?**

In 2015, all 22 countries and areas (countries) of the World Health Organization Eastern Mediterranean Region (EMR) pledged to achieve measles elimination by 2020. Despite success in several countries, most countries in the region still have not eliminated measles.

**What is added by this report?**

During 2019–2022, four EMR countries achieved measles elimination. However, regional coverage with the first and second measles vaccine doses remained at 82%–83% and 76%–78%, respectively, and surveillance performance deteriorated, in part because of effects of the COVID-19 pandemic. Annual regional measles incidence increased 68%, from 29.8 per 1 million population in 2019 to 50.0 in 2022.

**What are implications for public health practice?**

The regional measles elimination goal can be achieved through collaborative efforts to increase routine measles vaccination coverage, implement timely high-quality campaigns, and strengthen case-based surveillance.

## Abstract

In 2015, all 22 World Health Organization Eastern Mediterranean Region (EMR) countries and areas (countries) pledged to achieve measles elimination by 2020. Despite success in several countries, most countries in the region still have not eliminated measles. This report updates a previous report and describes progress toward measles elimination in EMR during 2019–2022. During that period, estimated regional coverage with the first and second doses of a measles-containing vaccine (MCV) was 82%–83% and 76%–78%, respectively. During 2019–2022, approximately 160 million children were vaccinated during national or subnational supplementary immunization activities. Reported confirmed regional measles incidence decreased from 29.8 cases per 1 million population in 2019 to 7.4 in 2020, but then increased 68%, to 50.0 in 2022 because of challenges providing immunization services and conducting surveillance during the COVID-19 pandemic. Surveillance indicators deteriorated in 11 (50%) of the 22 EMR countries. During 2019–2022, four countries in the region were verified as having achieved measles elimination, but other countries reported immunity gaps and increased measles incidence in 2022. To achieve measles elimination in EMR, national immunization programs, especially in those countries with high measles incidence, will need to continue to recover from the COVID-19 pandemic, increase overall vaccination coverage to close immunity gaps, and maintain high-quality disease surveillance.

## Introduction

In 2020, the World Health Assembly and partners endorsed the Immunization Agenda 2030 (IA2030) (*1*), a new global vision and strategy aiming to reach underimmunized and unimmunized children. This strategy builds on lessons learned from the Global Vaccine Action Plan (*2*) and emphasizes measles incidence and vaccination coverage as critical monitoring indicators for improving immunization services and strengthening primary health care. It also highlights the importance of rigorous measles surveillance to document immunity gaps and achieve ≥95% coverage with 2 doses of measles- and rubella-containing vaccines. The 2021–2030 Global Measles and Rubella Strategic Framework (*3*) and the 2021–2023 Global Measles Outbreak Strategic Response Plan (*4*) are also aligned with IA2030 to achieve measles and rubella elimination. In 2015, all 22 countries and areas (countries) of the World Health Organization (WHO) Eastern Mediterranean Region (EMR) endorsed the 2016–2020 Eastern Mediterranean Vaccine Action Plan and implemented country-specific strategic plans to achieve measles elimination (*5*). This report updates a previous report (*6*), describes the current epidemiology of measles in the EMR, and outlines what will be needed to reach the goal of regional measles elimination.

## Methods

### Immunization Activities

Data on administrative vaccination coverage* with the first and second doses of measles-containing vaccine (MCV) are reported each year from all EMR countries to WHO and UNICEF through the Joint Reporting Form (*7*). WHO and UNICEF use reported administrative coverage and available survey results to generate annual estimates of vaccination coverage through routine immunization services. Supplementary immunization activities (SIAs)^†^ are conducted in countries with low routine coverage and are an effective strategy for boosting population immunity. Data on SIAs are reported periodically by countries. SIA data and estimates of national and subnational vaccination coverage collected during 2019–2022 were reviewed for all EMR countries.

### Surveillance, Measles Incidence, and Measles Virus Genotypes

Case-based measles surveillance^§^ has been established in all EMR countries except Somalia. Case definitions for suspected measles include fever and rash, and measles cases are reported monthly using standardized reporting templates. Suspected measles cases are confirmed based on laboratory findings, an epidemiologic link to a confirmed case, or clinical criteria. All 22 national laboratories in the region provide serologic confirmation, and three perform genotyping of circulating viruses to facilitate monitoring of regional spread of measles virus genotypes (*8*). Sequence data are reported to the WHO global measles nucleotide surveillance database.^¶^ Case-based measles surveillance in EMR is monitored using seven important performance indicators**; four of these (timeliness and completeness of case investigations, adequacy of collection and testing of specimens, reporting of laboratory results within 4 days, and sensitivity of surveillance) are described in this report. Measles case data and surveillance performance indicators were summarized and analyzed descriptively. This activity was reviewed by CDC, deemed not research, and was conducted consistent with applicable federal law and CDC policy.^††^

### Regional Verification of Measles Elimination

The EMR Verification Commission for Measles and Rubella Elimination was established in February 2018 to evaluate the status of measles elimination in EMR countries based on documentation submitted annually by national verification committees (*9*). Reports of the commission were reviewed for this report.

## Results

### Immunization Activities

Estimated regional coverage with a first MCV dose (MCV1) remained stable at 82%–83% during 2019–2022 (Table 1); however, these were the lowest coverage levels since 2008. Regional coverage with a second MCV dose (MCV2) increased from 76% in 2019 to 78% in 2022. The number of countries achieving ≥95% national 2-dose MCV coverage increased from eight (36%) in 2019 to ten (45%) in 2021 but declined to 2019 levels in 2022 (Table 2). Only four (18%) countries achieved ≥95% MCV2 coverage in all districts during 2019–2022. During this period, 160 million persons were vaccinated during 40 SIAs conducted in 16 countries, with a weighted regional SIA coverage of 97% (Supplementary Table, https://stacks.cdc.gov/view/cdc/147631).

**TABLE 1:** Measles-containing vaccine vaccination schedule, estimated coverage with the first and second doses of measles-containing vaccine,* number of confirmed measles cases,^†^ and confirmed measles incidence,^§^ by country or area — World Health Organization Eastern Mediterranean Region, 2019–2022

Country/Area	MCV schedule^¶^		2019			2020			2021			2022		
			Coverage, %		No. of measles cases** (incidence)^§^	Coverage, %		No. of measles cases** (incidence)^§^	Coverage, %		No. of measles cases** (incidence)^§^	Coverage, %		No. of measles cases** (incidence)^§^
	First dose age, mos	Second dose age, mos	MCV1	MCV2		MCV1	MCV2		MCV1	MCV2		MCV1	MCV2	
**Total**	**—**	**—**	**83**	**76**	**22,549 (29.8)**	**83**	**77**	**7,630 (7.4)**	**82**	**77**	**16,860 (9.6)**	**83**	**78**	**39,266 (50.0)**
Afghanistan	9	18	64	41	212 (6.6)	66	43	512 (14.0)	63	44	2,916 (74.4)	68	49	5,090 (123.8)
Bahrain	12	18	99	99	0 (—)	99	99	0 (—)	99	99	0 (—)	99	99	18 (—)
Djibouti	9	15	83	81	0 (NR)	62	60	0 (NR)	50	48	0 (NR)	50	48	182 (162.4)
Egypt	12	18	95	94	0 (—)	94	94	0 (—)	96	96	0 (—)	96	96	14 (—)
Iran	12	18	99	98	0 (—)	99	98	1 (—)	99	98	104 (1.3)	99	98	231 (0.3)
Iraq	9	15	82	86	3,619 (31.7)	76	94	312 (8.4)	75	83	15 (0.5)	88	97	36 (0.8)
Jordan^††^	12	18	87	93	45 (4.5)	76	90	0 (—)	76	90	2 (0.2)	76	90	21 (1.9)
Kuwait^§§^	12	24	97	94	12 (2.9)	95	94	0 (—)	94	94	4 (0.7)	99	94	7 (1.6)
Lebanon^††^	12	18	82	63	1,046 (182.2)	74	64	15 (2.6)	67	59	5 (0.7)	67	59	86 (15.7)
Libya	12	18	73	72	188 (27.0)	73	72	20 (2.9)	73	72	5 (2.0)	73	72	13 (1.9)
Morocco	9	18	99	99	12 (0.3)	99	99	5 (0.1)	99	99	0 (—)	99	99	2 (0.1)
Oman	12	18	99	99	0 (—)	99	99	0 (—)	99	99	0 (—)	97	98	5 (—)
Pakistan	9	15	81	74	2,066 (9.7)	83	77	2,732 (12.1)	81	79	7,040 (31.3)	82	79	7,068 (30.0)
Palestine	12	18	99	99	228 (48.5)	99	99	833 (177.0)	98	99	0 (—)	97	93	0 (—)
Qatar	12	18	99	98	5 (1.8)	90	88	3 (1.1)	99	99	0 (—)	99	99	18 (1.9)
Saudi Arabia^††,§§^	12	18	95	96	1,035 (31.8)	96	96	29 (0.9)	98	97	13 (2.5)	98	98	149 (4.1)
Somalia	9	15	46	NA^¶¶^	4,482 (322.3)	46	NA^¶¶^	2,518 (198.8)	46	4	746 (59.5)	46	8	805 (45.8)
Sudan	9	18	90	74	3,555 (76.6)	86	68	354 (7.6)	81	63	627 (15.1)	81	63	1,272 (27.1)
Syria	12	18	65	54	27 (1.1)	59	53	14 (0.6)	59	53	11 (0.6)	41	38	217 (9.8)
Tunisia	12	18	98	97	4,669 (407.9)	98	96	11 (1.0)	95	98	2 (0.1)	95	98	10 (0.8)
United Arab Emirates^§§^	12	18	99	94	186 (20.4)	99	92	49 (5.5)	99	96	29 (3.5)	98	91	98 (10.4)
Yemen	9	18	67	46	1,162 (42.4)	68	46	222 (8.1)	71	52	5,341 (142.3)	73	56	23,924 (710.0)

**Abbreviations:** MCV = measles-containing vaccine; MCV1 = first MCV dose; MCV2 = second MCV dose; NA = not applicable; NR = not reported; WHO = World Health Organization; WUENIC = WHO and UNICEF estimates of national immunization coverage.

* According to WUENIC. For MCV1, among children aged 1 year or, if MCV1 is given at age ≥1 year, among children aged 24 months. For MCV2, among children at the recommended age for administration of MCV2, per the national immunization schedule. The WUENIC were last revised on July 15, 2023. https://immunizationdata.who.int/index.html

^†^ Includes cases confirmed by laboratory testing or epidemiologic linkage and clinically compatible cases. Clinically compatible cases met the WHO measles clinical case definition, had no adequate specimen collected, and could not be epidemiologically linked to a laboratory-confirmed case of measles.

^§^ Cases per 1 million population.

^¶^ MCV schedule is the 2022 schedule.

** Case totals based on WHO-UNICEF Joint Reporting Forms.

^††^ Additional 9-month dose provided nationally.

^§§^ Additional dose provided nationally at age 5–6 years (United Arab Emirates), 6 years (Saudi Arabia), or 12 years (Kuwait).

^¶¶^ Dose was not included in the vaccination schedule for that year.

**TABLE 2:** Countries and areas achieving measles immunization coverage, surveillance indicators, and incidence — World Health Organization Eastern Mediterranean Region, 2019–2022

Domain/Indicator	Indicator	Year, no. (%) of countries and areas meeting the indicator			
		2019	2020	2021	2022
**Routine immunization performance**					
National 2-dose MCV coverage	**≥95%**	8 (36)	7 (32)	10 (45)	8 (36)
Districts with ≥95% 2-dose MCV coverage (%)	**100%**	4 (18)	4 (18)	4 (18)	4 (18)
**Surveillance quality**					
Timeliness and completeness of suspected measles case investigation	**≥80%**	5 (23)	2 (9)	5 (23)	10 (45)
Percentage of suspected cases with adequate specimens collected, tested in a proficient laboratory	**≥80%**	21 (95)	21 (95)	14 (64)	17 (77)
Percentage of IgM laboratory results reported to national public health authorities within 4 days	**≥80%**	12 (55)	12 (55)	11 (50)	8 (36)
Annualized discarded (nonmeasles) case rate per 100,000 population	**≥2**	14 (64)	9 (41)	8 (36)	11 (50)
Endemic measles incidence*	**0**	3 (14)	5 (23)	6 (27)	5 (23)
	**>0 and <5**	7 (32)	9 (41)	11 (50)	8 (36)
	**≥5**	12 (55)	8 (36)	5 (23)	9 (41)

**Abbreviations:** IgM = immunoglobulin M; MCV = measles-containing vaccine.

* Cases per 1 million population.

### Surveillance, Measles Incidence, and Measles Virus Genotypes

During 2019–2022, the number of EMR countries that met the national target for surveillance sensitivity (two or more suspected cases per 100,000 population discarded as nonmeasles and nonrubella) declined from 14 (64%) in 2019 to 11 (50%) in 2022 (Table 2). During 2019–2022, the number of countries that achieved the target for timely and complete investigation of suspected measles cases increased from five (23%) in 2019 to 10 (45%) in 2022. The number that met the target of adequate specimens collected for laboratory testing decreased from 21 (95%) in 2019 to 17 (77%) in 2022, and the number with laboratory results reported within 4 days of specimen receipt decreased from 12 (55%) in 2019 to eight (36%) in 2022.

In EMR, the number of reported measles cases decreased by two thirds (66%) from 22,549 in 2019 to 7,630 in 2020, but then more than doubled (121% increase) to 16,860 in 2021, and then further increased (133%) from 2021 to 39,266 in 2022 (Table 1) (Figure). The increase in measles cases during 2021–2022 occurred primarily because of outbreaks in Afghanistan, Pakistan, and Yemen. Annual regional measles incidence (cases per 1 million population) decreased 75%, from 29.8 in 2019 to 7.4 in 2020, but increased 30% to 9.6 in 2021 and further increased by 421% from 9.6 in 2021 to 50.0 in 2022. The number and percentage of countries reporting a measles incidence of ≥5 per million decreased from 12 (55%) in 2019 to five (23%) in 2021 but increased to nine (41%) in 2022 (Table 2). During 2019–2022, two circulating measles genotypes were detected among 1,345 specimens sequenced in EMR, including 1,298 (97%) of genotype B3 in 16 countries and 44 (3%) of genotype D8 in seven countries.

**FIGURE:**
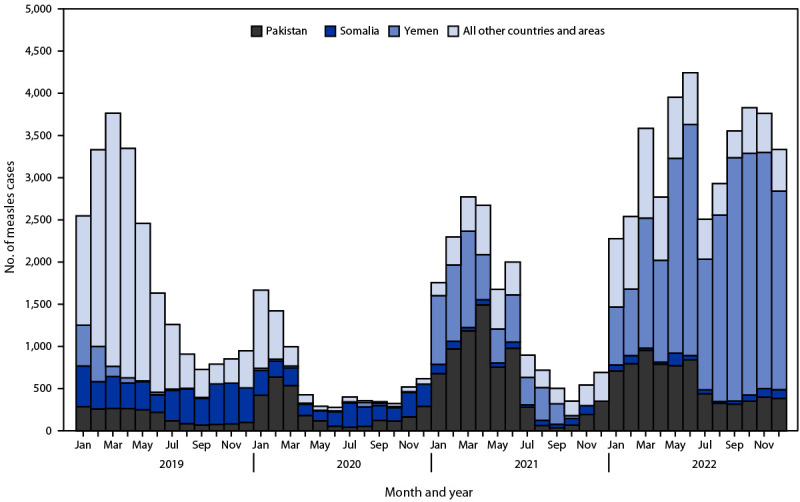
Confirmed measles cases,* by month and year of rash onset — World Health Organization Eastern Mediterranean Region, 2019–2022 **Abbreviation:** WHO = World Health Organization. * Confirmed and clinically compatible measles cases reported to WHO by countries and areas. A case of measles was laboratory-confirmed when measles-specific immunoglobulin M antibodies were detected in serum, or measles-specific RNA was detected by polymerase chain reaction testing in a person who was not vaccinated during the 30 days before rash onset. A case of measles lacked serologic confirmation but was confirmed by epidemiologic linkage when linked in time and place to a case of laboratory-confirmed measles. During 2013–2019, a case of measles meeting the WHO case definition but without a specimen collected could be reported as clinically compatible.

### Regional Verification of Measles Elimination

The Verification Commission for Measles and Rubella Elimination convened four times during 2019–2022. By the end of 2019, three (14%) EMR countries (Bahrain, Iran, and Oman) were verified as having achieved measles elimination, and in 2021, Egypt was also verified. By the end of 2022, these four countries were confirmed to have maintained measles elimination (Bahrain, Iran, and Oman for 3 years, and Egypt for 1 year).

## Discussion

Despite the regional challenges, 11 (50%) of the 22 EMR countries are progressing toward measles elimination, and elimination has been verified in four (18%) countries. Important characteristics of these four countries include having health ministries committed to measles elimination, sustained high (≥95%) immunization coverage, and strong surveillance systems and laboratory support. An additional seven countries (Kuwait, Morocco, Palestine, Qatar, Saudi Arabia, Tunisia, and United Arab Emirates) are near elimination based on high measles immunization coverage, low measles incidence, and high-quality surveillance. 

However, eleven (50%) EMR countries are experiencing conflicts or humanitarian crises that prevent immunization system strengthening and prioritization of measles elimination. These challenges have resulted in underperforming immunization programs, leading to measles immunity gaps. Of particular concern are undervaccinated children (those who have not received 2 MCV doses) and unvaccinated children (those who have not received any MCV or other vaccine doses). These children generally reside in hard-to-reach locations and experience conflict-related insecurity, misinformation, and underperforming vaccination campaigns. Communities with large numbers of undervaccinated and unvaccinated children are at increased risk for measles outbreaks and measles-related deaths.

Among countries with fragile health systems and measles immunity gaps, increasing MCV1 and MCV2 coverage and conducting high-quality SIAs with a focus on reaching populations at high risk, particularly those living in areas with civil strife, are needed. A strong global partnership is needed to work together to build measles immunity and prevent measles mortality through routine immunization services and preventive SIAs. Conducting preventive SIAs in areas with complex humanitarian emergencies, however, requires strong coordination among global and local partners and stakeholders in all facets of vaccination campaigns for these SIAs to be successful.

In addition, conducting surveillance in areas with political instability, insecurity, and a complex operating environment is challenging. This challenge is often compounded by the absence of strong data systems that prevent efficient reporting, analysis, and use of data for action. As a result, responses to outbreaks might not be timely and effective. Further, poor immunization and surveillance data quality in many countries hamper their ability to assess measles immunity gaps and plan timely campaigns to prevent outbreaks. Overcoming these challenges will require partnerships and support at global, national, and local levels to optimize surveillance and ensure rapid detection and response to measles cases and outbreaks.

### Limitations

The findings in this report are subject to at least three limitations. First, administrative coverage might be inaccurate because data quality and consistency vary substantially among different countries. Second, measles cases might be underestimated because not all measles patients seek health care, not all cases are reported or investigated, and measles surveillance quality varies among countries. Finally, this report did not consider measles mortality because few EMR countries monitored or reported measles-related deaths.

### Implications for Public Health Practice

Measles cases increased in EMR after the COVID-19 pandemic because of inadequate vaccination coverage, resulting in widening of immunity gaps, and declining measles surveillance performance. Routine measles vaccination activities and SIA implementation need to continue or increase in those countries, and efforts to conduct timely case-based surveillance and laboratory testing need to resume. Supporting countries with fragile health systems and reaching undervaccinated and unvaccinated children with ≥2 MCV doses are critical to achieving regional measles elimination.

## Footnotes

* Administrative vaccination coverage is the number of vaccine doses administered divided by the estimated target population.

† Two SIA approaches are used: 1) a nationwide catch-up SIA, targeting wide age groups to eliminate population susceptibility, and 2) periodic follow-up SIAs every 2–4 years, targeting all children born since the last SIA. The exact age range for follow-up SIAs depends on the age-specific measles incidence, coverage with routine MCV, and the interval since the last SIA.

§ Case-based measles surveillance includes individual case investigation and blood specimen collection for laboratory testing.

¶
https://who-gmrln.org/means2

** Important surveillance performance indicators monitored at the regional level include 1) reporting rate of discarded nonmeasles nonrubella cases at the national level (two cases per 100,000 population per year); 2) proportion of suspected measles and rubella cases that have had an adequate investigation initiated within 48 hours of notification (80% of suspected cases); 3) proportion of suspected cases with adequate specimens collected for detecting acute measles or rubella infection collected and tested in a proficient laboratory (80%); 4) proportion of specimens received at the laboratory within 5 days of collection (80%); 5) proportion of immunoglobulin M results reported to national public health authorities by the laboratory within 4 days of specimen receipt (80%); 6) proportion of laboratory-confirmed measles outbreaks with specimens adequate for detecting measles virus collected and tested in an accredited laboratory (80%); and 7) percentage of confirmed cases for which source of transmission is classified as endemic, imported, or importation-related (80%).

†† 45 C.F.R. part 46.102(l)(2), 21 C.F.R. part 56; 42 U.S.C. Sect. 241(d); 5 U.S.C. Sect. 552a; 44 U.S.C. Sect.3501 et seq.
